# TrailMap: Pheromone-Based Adaptive Peer Matching for Sustainable Online Support Communities

**DOI:** 10.3390/biomimetics10100658

**Published:** 2025-10-01

**Authors:** Harold Ngabo-Woods, Larisa Dunai, Isabel Seguí Verdú, Dinu Turcanu

**Affiliations:** 1 Universitat Politècnica de València, 46022 Valencia, Spain; hawooka@doctor.upv.es; 2 BGDV-Hanbo Research, 1 KN 78 St, Kigali, Rwanda; 3 Department of Graphical Engineering, Universitat Politècnica de València, 46022 Valencia, Spain; 4 Department of Software Engineering and Automatics, Technical University of Moldova, MD-2004 Chisinau, Moldova; dinu.turcanu@adm.utm.md

**Keywords:** ant colony optimisation, biomimetic routing, digital peer support, mental health HCI, stigmergy, load balancing, digital therapeutics (DTx), participation inequality

## Abstract

Online peer support platforms are vital, scalable resources for mental health, yet their effectiveness is frequently undermined by inefficient user matching, severe participation inequality, and subsequent “super-helper” burnout. This study introduces TrailMap, a novel peer-matching algorithm inspired by the decentralised foraging strategies of ant colonies. By treating user interactions as paths that gain or lose “pheromone” based on helpfulness ratings, the system enables the community to collectively and adaptively identify its most effective helpers. A two-phase validation study was conducted. First, an agent-based simulation demonstrated that TrailMap reduced the mean time to a helpful response by over 70% and improved workload equity compared to random routing. Second, a four-week randomised controlled pilot study with human participants confirmed these gains, showing a 76% reduction in median wait time and significantly higher perceived helpfulness ratings. The findings suggest that by balancing the workload, TrailMap enhances not only the efficiency but also the socio-technical sustainability of online support communities. TrailMap provides a practical, nature-inspired method for building more resilient and equitable online support communities, enhancing access to effective mental health support.

## 1. Introduction

### 1.1. The Scalability and Sustainability Crisis in Digital Peer Support

The demand for mental health services has outstripped the supply of qualified professionals, a chasm widened by recent global events that not only increased the need for care [1] but also intensified pre-existing digital inequalities, further isolating vulnerable groups who depend on online support systems [2]. Digital mental health interventions (DMHIs) and digital therapeutics (DTx) have emerged as primary strategies to democratise access to care, offering scalable and accessible solutions [3,4]. Within this landscape, online peer support platforms have become a critical resource, providing users with immediate and destigmatised support from individuals with shared lived experiences [5,6,7]. These communities offer a sense of belonging and validation, which are instrumental for recovery [8,9].

However, the long-term viability and effectiveness of these platforms are threatened by a tripartite crisis of scalability and sustainability rooted in their underlying architecture. The first is inefficient matching. Most platforms rely on indiscriminate broadcast methods or simple chronological queues to connect help-seekers to providers. These non-adaptive routing mechanisms often result in long response delays, low-quality initial interactions, and high rates of user attrition as help-seekers become frustrated and disengaged [5,10].

The second challenge is participation inequality. Online communities are consistently governed by a power-law distribution of activity, often termed the ’1% rule’ or the ’90-9-1 rule’, where a small fraction of users account for most content creation and interaction [11,12]. In support communities, this manifests as the ’super-helper’ phenomenon, where a small cohort of users, often driven by a combination of altruism, domain experience, and high intrinsic motivation, handles a disproportionate volume of support requests [13,14]. Although these individuals constitute the essential foundation of the community, the system’s dependence on them introduces a vulnerable, single point of failure.

This systemic vulnerability culminates in helpful burnout, which is the most critical challenge to the long-term sustainability of these platforms. This syndrome is defined by emotional exhaustion, depersonalisation, and a reduced sense of accomplishment and is a direct consequence of the immense and unequally distributed workload placed upon these ’super-helpers’ [15]. This phenomenon, although specific to the informal dynamics of these communities, is comparable to the extensively documented syndromes of occupational impairment observed among formal healthcare professionals [16]. When these vital contributors inevitably reduce their activity or leave the community, the platform’s collective capacity and quality of support are severely degraded [17,18,19]. This creates a vicious cycle of decaying community health. Therefore, the core problem is not merely optimising efficiency but ensuring the socio-technical sustainability of the entire support ecosystem. An intelligent matching algorithm must not only connect users quickly but also regulate the community’s social dynamics to foster resilience and prevent collapse.

### 1.2. Biomimicry for Decentralised Optimisation: The Ant Colony Metaphor

Nature provides elegant solutions to complex decentralised optimisation problems [20]. A canonical example is the foraging behaviour of ant colonies. Individual ants, operating with simple rules and no central oversight, collectively discover the shortest paths between their nests and food sources [21]. This is achieved through a mechanism known as stigmergy, a form of indirect communication where individuals interact by modifying their shared environment [22,23].

As an ant traverses a path, it deposits a volatile chemical substance called a pheromone. The subsequent ants are probabilistically biased to follow paths with stronger pheromone concentrations. Because shorter paths can be traversed more frequently in a given time period, they accumulate pheromones at a faster rate, creating a positive feedback loop that rapidly reinforces the optimal route [21]. Concurrently, the pheromones evaporate over time. This decay process is crucial; it allows the colony to ’forget’ old or suboptimal paths and adapt to a dynamic environment, such as when a food source is depleted or a new, better one appears [24].

This robust, adaptive, and decentralised system has been successfully abstracted into a powerful class of computational metaheuristics known as Ant Colony Optimisation (ACO) [24]. First proposed by Dorigo, Maniezzo, and Colorni [21], ACO has been applied to a wide range of complex combinatorial problems, from the “Travelling Salesman Problem” to dynamic network routing [25,26,27,28,29]. The core principles of ACO include probabilistic path selection based on trail intensity and time-based trail evaporation, which provide a compelling framework for designing self-organising systems [24].

### 1.3. The TrailMap Hypothesis and Contribution

This study proposes that the principles of ACO can be abstracted to address the sustainability crisis in digital peer support. The proposed algorithm, TrailMap, treats the peer support community as a dynamic graph and the matching process as a routing problem. The core features of ACO are particularly relevant for counteracting the specific failure modes of these online communities. The evaporation mechanism is fundamental to the system’s adaptivity; by ensuring trails decay over time, it allows the system to “forget” paths leading to helpers who have become inactive or burned out, thus preventing a static lock-in on outdated information. Simultaneously, stochastic path selection ensures that new or less-frequent helpers have a chance to be discovered, counteracting the entrenchment of a small “super-helper” elite and promoting a more equitable distribution of the support load.

Our central contribution, therefore, is to reframe this challenge from one of static matching to one of adaptive routing. Unlike non-adaptive routing systems (e.g., chronological queues), which are blind to the dynamic social qualities of a community, TrailMap is inherently adaptive, adjusting its routing in response to real-time, socially constructed data. Furthermore, unlike conventional recommender systems that typically focus on preference prediction within a static item catalogue, TrailMap is designed as a dynamic resource allocation system. It manages a network of human helpers with fluctuating availability and capacity, optimising not just for immediate match quality but for the long-term sustainability and equity of the entire support ecosystem. This positions TrailMap as a novel form of societal algorithmic mediation aimed at fostering resilient and self-regulating digital communities. For instance, whereas a conventional recommender system mediates a user’s interaction with a static catalogue of content (e.g., movies or products), TrailMap mediates social interactions between users, actively managing the distribution of support requests to prevent helper burnout and ensure the community’s long-term viability.

The innovation of this approach is fourfold, as follows:

1. **Novel problem framing:** We are the first to frame the challenge of online support sustainability as a dynamic, decentralised resource allocation problem solvable with a stigmergic approach;
2. **A novel pheromone mechanism:** Our pheromone deposition rule $\left(∆\tau_{sh}=R_{h}-3\right)$ is a specific implementation that directly translates subjective human feedback into a mechanism for both positive and negative reinforcement, allowing the community to self-police and suppress unhelpful actors;
3. **A novel conceptual contribution:** We demonstrate how a classic computational challenge—the ‘cold start’ problem—is transformed into a feature for social sustainability and community renewal (discussed in Section 5.1);
4. **A novel empirical validation:** The two-phase study, combining agent-based simulation with a human-centered RCT, provides a rare and robust validation of a biomimetic algorithm in a real-world social context.

We hypothesise that by implementing this pheromone-based routing algorithm, we can create a self-regulating digital ecosystem that is not only more efficient but also more sustainable. Compared to standard random routing, TrailMap will achieve the following:

- Significantly decrease the wait time for a help-seeker to receive a high-quality response;Dynamically identify and prioritise highly rated helpers, improving the overall perceived quality of support;
- Distribute the load of responding more equitably, mitigating the structural conditions that lead to super-helper burnout.

This paper presents the formal specification of the TrailMap algorithm and validates these hypotheses through a two-phase study. First, an agent-based simulation was used to analyse the algorithm’s dynamics and optimise its key parameter (pheromone evaporation rate). Second, a human-centered, randomised controlled pilot study was conducted to evaluate its efficacy and user experience in a real-world setting. Through this study, we demonstrate that the biomimetic principles embodied in TrailMap can successfully address key sustainability challenges in peer support, thus offering a powerful new approach for designing the next generation of digital therapeutics.

### 1.4. Positioning TrailMap Within Evolutionary Optimisation

Ant Colony Optimisation (ACO) belongs to a broader class of nature-inspired metaheuristics used to solve complex optimisation problems. It is important to position our choice of ACO in relation to other prominent evolutionary algorithms, such as genetic algorithms (GAs), particle swarm optimisation (PSO), and simulated annealing (SA).

Genetic algorithms (GAs) are inspired by natural selection, using operators like crossover and mutation to evolve a population of candidate solutions (‘chromosomes’) over generations. GAs have been successfully applied to network load balancing but often rely on a centralised fitness function to evaluate the entire system state [30,31,32,33].

Particle swarm optimisation (PSO) models the social behaviour of bird flocks, where individual ‘particles’ adjust their trajectory based on their own best-known position and the best-known position of the entire swarm. PSO is powerful for continuous optimisation problems and has been used to calibrate agent-based models and fine-tune recommender systems [34,35,36].

Simulated annealing (SA) is a probabilistic technique modelled on the annealing process in metallurgy, where a material is heated and slowly cooled to reach a minimum energy state. It is effective for avoiding local optima and has been applied to dynamic routing problems [37,38,39].

While these methods are powerful, we selected ACO because its core mechanism—stigmergy—is a particularly strong analogue for the socio-technical dynamics we aimed to model. Unlike GAs or global-best PSOs that often require a holistic view of the solution space, ACO relies on decentralised, asynchronous, and local modifications to a shared environment (the pheromone trails). This bottom-up process mirrors how a real online community builds a collective sense of reputation and trust over time. The explicit mechanisms of pheromone deposition and evaporation provide a more direct and interpretable model for social learning, memory, and forgetting than the more abstract operators of GAs or the trajectory-based updates of PSO. Our contribution, therefore, is not just to optimise a network, but to leverage the specific biomimetic properties of ACO to regulate a human social system. Recent work has also highlighted the power of hybrid approaches, such as combining GAs with agent-based models [40], which represent promising future directions.

## 2. The TrailMap Algorithm

### 2.1. System Model

The peer support community is modelled as a dynamic, directed graph *G* = (*U*, *E*), where *U* is the set of all registered users (nodes) and *E* represents the set of all possible seeker–helper interactions (edges). Each edge (*s*, *h*) from a help-seeker (user *s*) to a help-provider (user *h*) possesses an associated weight, $\tau_{sh}$, which represents the level of ‘digital pheromone’ on that potential interaction path. This pheromone value is dynamic and updated through the processes of deposition and evaporation.

### 2.2. Pheromone Deposition (Trail Reinforcement)

Direct user feedback drives the mechanism for reinforcing successful support pathways. Following an interaction, the help-seeker (user *s*) is prompted to provide a perceived helpfulness rating ${(R}_{h})$ for the support received from the helper (user *h*). This rating is captured on a 5-point Likert scale (1 = very unhelpful, 2 = unhelpful, 3 = neutral, 4 = helpful, 5 = very helpful).

The amount of pheromone deposited, ${∆\tau }_{sh}$, on the edge from any seeker to a specific helper (user *h*) is calculated based on this rating. To allow the system to learn from both positive and negative experiences, the deposition rule is centered around the neutral rating, expressed as follows:

(1) $$∆\tau_{sh}={(R}_{h}-3)$$

This formulation is critical for the ethical function of the algorithm. A rating of 4 or 5 deposits a positive pheromone, strengthening the trail and increasing the probability of future routing to that helper. A neutral rating of 3 deposits no pheromone, leaving the trail unchanged. Crucially, a rating of 1 or 2 deposits a negative pheromone, effectively creating a negative pheromone trail that actively discourages the algorithm from routing future seekers to a helper who has been rated as unhelpful or harmful. The total pheromone on the path to a helper, $\tau_{h}$, is the sum of all depositions from all interactions they have had.

### 2.3. Path Selection (Probabilistic Routing)

When a new seeker (user *s*) submits a request for support, the system first identifies the set of currently available helpers, $N_{h}$. In our implementation, availability is defined as any registered user who is currently logged into the platform and is not already engaged in active support interaction.

The probability (*P*) of routing the request to any specific available helper $\left(u_{h}\in N_{h}\right)\;$is determined by a stochastic decision rule that balances exploitation (favouring helpers with strong pheromone trails) and exploration (sampling less-established helpers). This probability was calculated using the standard ACO selection formula [21]:

(2) $$P\left(s,\;h\right)=\frac{{(\tau }_{h}^{\alpha }+\tau_{min})}{\sum k\in N\text{\_}h\;(\tau_{k}^{\alpha }+\tau_{min})}$$

where $\tau_{h}$ is the total pheromone level associated with helper $u_{h}$. α is a parameter that controls the influence of the pheromone trail (heuristic importance). A higher α makes the selection process more greedy, whereas α = 0 results in purely random selection. In this study, α was set to 1. $\tau_{min}$ is a small positive constant (set to 0.1 in our implementation) that ensures that even helpers with zero or negative total pheromones have a nonzero probability of being selected. This is essential for facilitating exploration, allowing new helpers to receive their first request, and preventing the system from prematurely converging on a small set of established helpers.

### 2.4. Pheromone Evaporation (Trail Decay)

To ensure that the system remains adaptive to the dynamic nature of the community, such as a helpful user becoming inactive, a user’s helpfulness changing over time, or the emergence of new, effective helpers, all pheromone trails must decay over time. This ‘forgetting’ mechanism is a cornerstone of Ant Colony Optimisation, preventing the system from becoming permanently locked into outdated pathways and allowing it to ‘forget’ suboptimal solutions in a changing environment [24]. This feature makes the algorithm suitable for dynamic, rather than static, optimisation problems.

At discrete time intervals (∆*t*) (in our implementation, every hour), every pheromone trail $\tau_{h}$ associated with a helper $u_{h}$ is updated according to the canonical exponential decay rule used in the ACO, expressed as follows:

(3) $$\tau_{h}\left(t+\;∆t\right)=\left(1-\rho \right)\;\tau_{h}\left(t\right)$$

where $\rho \in \left(1,0\right)$ is the pheromone evaporation rate. This parameter is the most critical lever for tuning the memory and responsiveness of a system. Mathematically, it represents the decay constant in an exponential function governing the half-life of the community’s collective memory of the helper’s effectiveness. A high value of *ρ* (e.g., approaching 1) causes trails to decay very quickly, making the system highly sensitive to the most recent feedback, but potentially unstable and unable to build strong, lasting trails to consistently good helpers. Conversely, a low value of *ρ* (e.g., approaching 0) builds strong, stable trails but adapts very slowly to changes, risking the routing of seekers to helpers who are no longer active or effective.

The relationship between the hourly evaporation rate ρ and the more intuitive metric of pheromone half-life $T_{1/2}$ (the time in hours for a trail’s value to be reduced by half) is given by the formula $\rho =1-0.5^{{(1/T}_{1/2})}.$ The optimal value for this parameter, which strikes a balance between adaptability and stability in our specific socio-technical context, was determined experimentally in our agent-based simulation (see Section 4.1) to be a half-life of 48 h.

The choice of this parameter is not merely a technical optimisation; it is an implicit ethical and social design choice. It encodes a value judgment about how long the community should “trust” a helper’s past performance versus how quickly it should adapt to new information and give new or intermittent helpers an opportunity to contribute. A 48 h half-life was found to balance the rewards of established trusted helpers with the necessary dynamism to foster a resilient and inclusive community.

**Figure 1:**
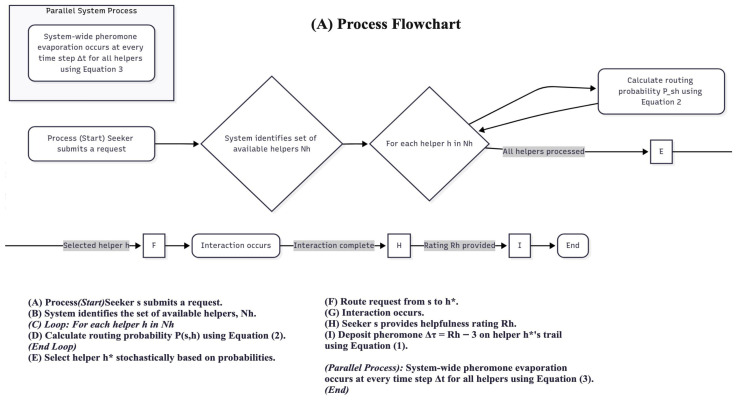

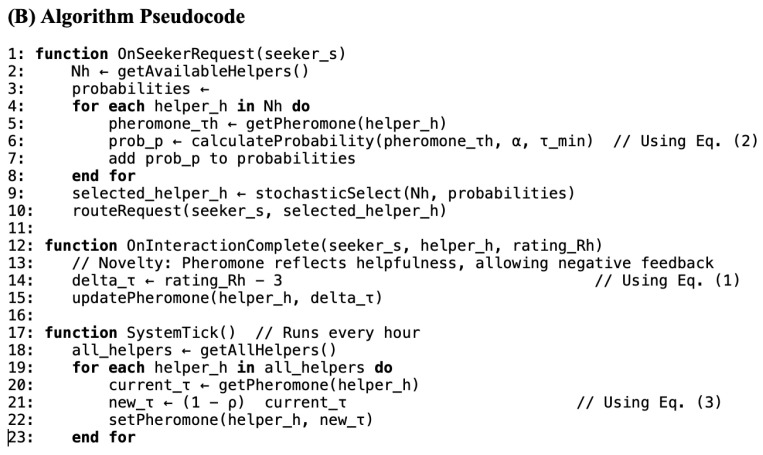
(**A**) Process flowchart and (**B**) pseudocode for the TrailMap algorithm.

The complete operational flow of the TrailMap algorithm, from a user’s request to the continuous updating of the pheromone landscape, is summarised in Figure 1.

## 3. Materials and Methods

### 3.1. Study 1: Agent-Based Simulation

**Objective:** The primary objectives of the agent-based simulation were (1) to test the fundamental dynamics of the TrailMap algorithm against a control under controlled conditions that included several idealised assumptions, such as constant rates of user activity and static, predefined helper attributes, and (2) to perform a sensitivity analysis to identify an optimal pheromone evaporation rate (*ρ*) that balances routing efficiency with load equity.

**Framework:** The simulation was developed in Python 3.9, utilising the Mesa 1.2.1 library, a standard framework for agent-based modelling. The simulation was run for a period of 30 virtual days (720 h), with a time step of 1 h for pheromone evaporation calculations.

**Model Parameters:** The simulation instantiated a community of N = 1000 agents, governed by the formal parameters detailed in Table 1.

- **Roles:** At any given time, a fixed proportion of agents were designated as active seekers (20%, *n* = 200) or potential helpers (80%, *n* = 800). Seeker agents generated new support requests at a fixed rate.
- **Helper Characteristics:** To model a realistic population, each helper agent was assigned an intrinsic, latent ‘helpfulness’ score, a value drawn from a Beta distribution with parameters α = 2 and β = 5. This created a right-skewed distribution, representing a community with many moderately helpful individuals and a smaller number of exceptionally effective helpers.
- **Interaction Model:** When a seeker agent receives a response from a helper agent, the helpfulness rating it provides ${(R}_{h})$ is stochastically generated. The rating is drawn from a discrete distribution, the mean of which is the helper’s latent helpfulness score, with Gaussian noise added to simulate the subjective and variable nature of human perception.

**Conditions:** Two experimental conditions were simulated.

- **TrailMap:** Help-seekers were matched to available helpers using the full TrailMap algorithm, as described in Section 2.
- **Random Routing (Control):** Help-seekers are matched to any available helper with a uniform probability, simulating the baseline condition in many existing peer support platforms.
- **Sensitivity Analysis:** To determine the optimal evaporation rate, the TrailMap condition was simulated across five distinct pheromone half-lives: 6, 12, 24, 48, and 72 h. The half-life ($T_{1/2}$) was related to the hourly evaporation rate (*ρ*) according to the formula $\rho =1-0.5^{{(1/T}_{1/2})}.$

**Metrics:**

- **Mean Time to Helpful Response (TTHR):** The primary efficiency metric, defined as the average time in minutes from the moment a seeker agent generates a request until it receives a response that it rates as ≥4 (‘Helpful’ or ‘Very Helpful’).
- **Load Distribution (Gini Coefficient):** The primary equity metric. The Gini coefficient, a standard measure of statistical dispersion typically used for income inequality, was applied to the distribution of requests handled per helper agent over a 30-day simulation [41,42]. Its application to measure heterogeneity and load imbalance in complex networks is well established [29,43,44,45]. A Gini coefficient of 0 indicates perfect equality (all helpers handle an identical number of requests), whereas a coefficient of 1 indicates perfect inequality (one helper handles all requests). This metric provides a robust, single-value measure of workload concentration and the “super-helper” effect.

### 3.2. Study 2: Human-Centered Pilot Study

**Objective:** This pilot study was designed to validate the findings of an agent-based simulation in a real-world context with human participants. The goals were to measure the algorithm’s impact on wait times and perceived support quality, and to gather qualitative feedback on the user experience.

**Ethical Approval:** This study was conducted in accordance with the Declaration of Helsinki. Ethical approval was granted by the Ethics Committee of the Blithe Research Initiative (BRI) (BRI.581314.18291/3/6). All participants provided informed digital consent prior to enrolment, which detailed the study’s purpose, duration, data handling procedures, and their right to withdraw at any time.

**Participants and Recruitment:** We recruited 71 volunteers from the university community through mailing lists and advertisements. An initial screening ensured that participants were over 18 years old and not in acute psychological distress. Participants were explicitly informed that the platform was a prototype for general wellness and stress management support and was not a substitute for professional clinical care or crisis intervention services. Demographic data were collected via a baseline questionnaire to ensure that the randomisation process resulted in comparable groups (see Table 2).

**Procedure:** A four-week, double-blind, randomised controlled trial was conducted. Participants were randomly assigned to one of the following two conditions using a computer-generated sequence:

- **TrailMap Group (*n* = 36):** Participants used a web-based peer support application where backend matching was governed by the TrailMap algorithm using the optimal 48 h pheromone half-life identified in Study 1.
- **Control Group (*n* = 35):** Participants used an identical user interface, but the backend matching algorithm was purely random, connecting seekers to any available online helper with a uniform probability. Participants in both groups were instructed to log in to the platform at least three times per week and to engage as they saw fit, either by posting requests for support or responding to requests from others.

**Measures:**

- **Primary Outcome (Efficiency):** Median wait time to first response, measured in minutes. This was calculated from server-side timestamps from the moment a request was submitted to the moment the first response was posted. System logs were used for this purpose.
- **Secondary Outcome (Quality):** Mean helpfulness rating. After each interaction, seekers were prompted to rate the helpfulness of the response(s) they received on the same 1–5 Likert scale used in the algorithm’s deposition rule.
- **Qualitative Feedback:** At the conclusion of the four-week study, all participants were invited to complete a brief open-ended survey. The survey included questions about their overall experience, perception of the matching process, level of trust in the system, and any suggestions for improvement.
- **Data Analysis:** All statistical analyses were conducted using R (version 4.2.2).

The wait-time data were heavily right-skewed, violating the assumption of normality. Therefore, a non-parametric Mann–Whitney U test was used to compare the median wait times between the two groups. Helpfulness ratings were approximately normally distributed and compared using an independent sample *t*-test. The significance level (alpha) for all tests was set at α = 0.05. Qualitative data from the open-ended surveys were analysed using an inductive thematic analysis approach to identify recurring patterns and themes in participant experiences [46].

## 4. Results

### 4.1. Simulation Findings: Efficiency and Equity

The agent-based simulation demonstrated that the TrailMap algorithm configured with the optimal 48 h evaporation half-life outperformed the random routing control. As summarised in Table 3, it reduced the mean time to helpful response (TTHR) from 84 min to 24 min, representing a 71.4% reduction, and it lowered the Gini coefficient of load imbalance from 0.58 to 0.34. These results signify an improvement in both system efficiency and equity. The sharp decrease in TTHR indicates that seekers were connected to high-quality support far more quickly, while the reduction in the Gini coefficient demonstrates a shift away from the significant inequality observed in the control group towards a far more balanced distribution of the support load across the entire helper agent population.

**Figure 2:**
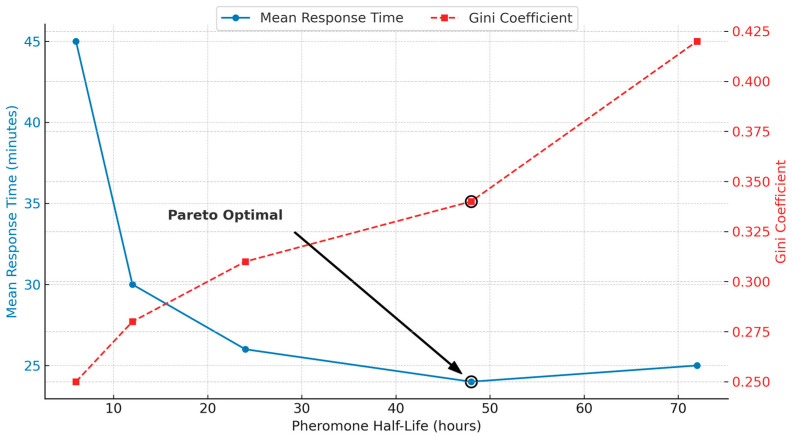
Sensitivity Analysis of Pheromone Evaporation Half-Life. Tradeoff between mean response time and load equity (Gini coefficient). The 48 h evaporation half-life provides a near-minimal response time while maintaining a significantly lower Gini coefficient than longer half-lives, identifying it as the optimal parameter for balancing efficiency and sustainability.

The sensitivity analysis for the pheromone evaporation rate demonstrated a distinct trade-off between system responsiveness and trail stability, as illustrated in Figure 2. Shorter half-lives (e.g., 6 h) were highly adaptive but struggled to build strong pheromone trails, resulting in longer response times (approx. 42 min), despite achieving a very low Gini coefficient (approx. 0.33). Conversely, longer half-lives (e.g., 72 h) built very strong trails but were slow to adapt to changes in helper agent availability, leading to an increase in both response time (approx. 29 min) and a significantly higher load concentration (Gini coefficient 0.42). The 48 h half-life emerged as the Pareto optimal parameter, achieving a minimal response time of 24 min while maintaining a low Gini coefficient of 0.34.

**Figure 3:**
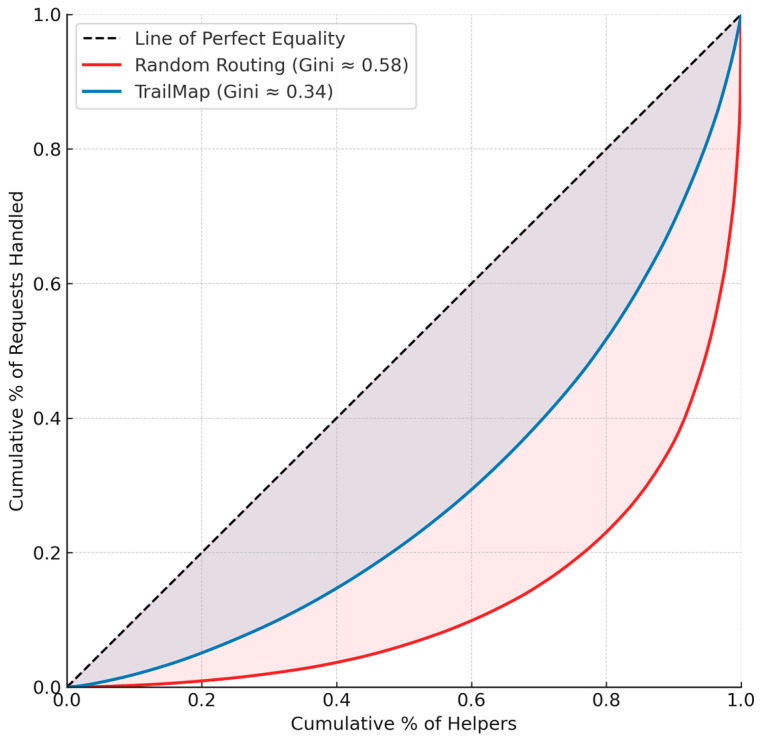
Lorenz Curves for Simulated Load Distribution. Lorenz curves illustrating the cumulative distribution of support requests handled by helper agents in the simulation. The TrailMap algorithm results in a distribution that is significantly closer to the line of perfect equality than random routing, providing strong visual evidence of its ability to mitigate load imbalance.

The impact of TrailMap on the load distribution is further visualised by the Lorenz curves in Figure 3. The curve for the random routing condition deviates significantly from the line of perfect equality, showing that the top 20% of helpers handled over 60% of the requests. The TrailMap curve is substantially closer to the line of equality, illustrating a much healthier distribution, where the workload was shared more broadly across the community.

### 4.2. Pilot Study Validation: Real-World Performance

A four-week pilot study with 71 human participants successfully validated the core findings of the simulation. The quantitative and statistical results are summarised in Table 4.

**Figure 4:**
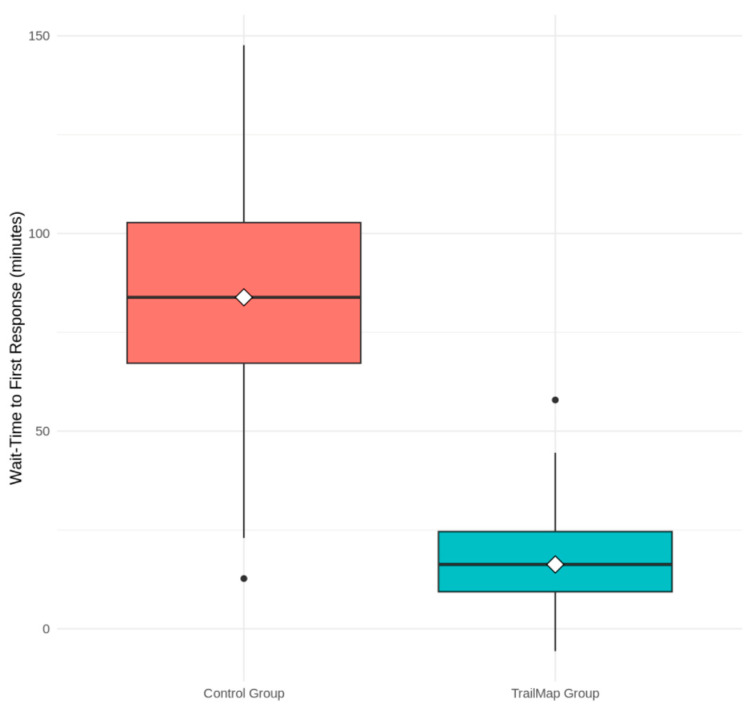
Comparison of Median Wait Times in the Pilot Study. Distribution of wait times to first response for the control group (random routing) and TrailMap group (pheromone routing). The TrailMap algorithm resulted in significantly lower and less variable wait times (*p* < 0.001).

Participants in the TrailMap group experienced substantially shorter wait times for their first response. As shown in the boxplot in Figure 4, the median wait time for the TrailMap group was 19 min (Interquartile Range = 12–31). This was a 76.8% reduction from the control group’s median wait time of 82 min (IQR = 45–124). A Mann–Whitney U test confirmed that this substantial difference was statistically significant (U = 217, *p* < 0.001).

**Figure 5:**
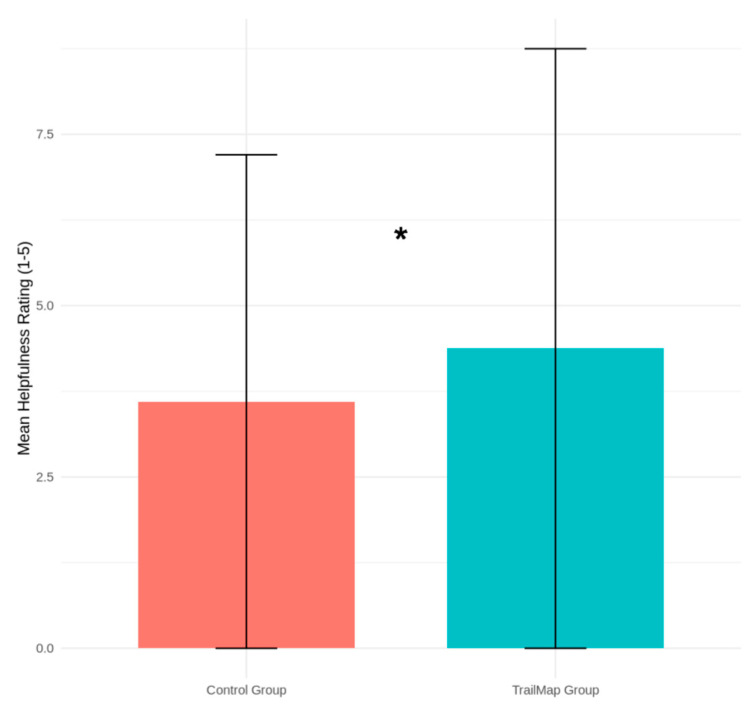
Comparison of Mean Helpfulness Ratings in the Pilot Study. Mean helpfulness ratings for interactions in the control and TrailMap groups. Interactions mediated by the TrailMap algorithm were rated as significantly more helpful by users (*p* < 0.001). The asterisk (*) indicates a statistically significant difference (*p* <.001).

The perceived quality of support received was also significantly higher in the TrailMap group. The mean helpfulness rating provided by seekers in the TrailMap group was 4.31 (SD = 0.68) compared to 3.45 (SD = 1.02) in the control group. An independent samples *t*-test found this difference to be statistically significant (t(69) = 4.51, *p* < 0.001). This comparison is shown in Figure 5.

**Qualitative Feedback:** Thematic analysis of the post-study survey responses revealed three distinct themes differentiating the user experience between the groups.

**Figure 6:**
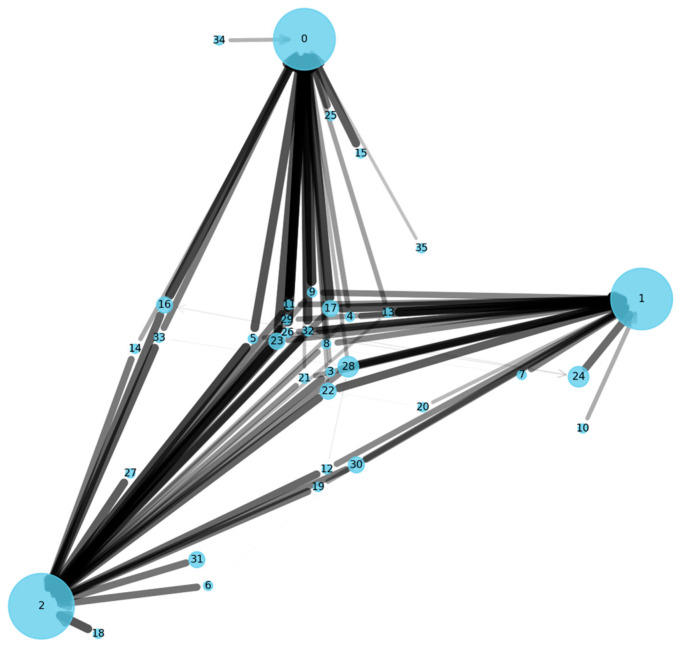
Interaction Graph from the TrailMap Group. Visualisation of the user interaction graph from the TrailMap group after four weeks. The node size represents the volume of requests handled. The edge thickness represents the pheromone strength. The graph shows the emergence of highly rated ’mentor’ nodes that attract strong, positive trails from many seekers, indicating the algorithm’s success in identifying and leveraging effective helpers.

- **Perceived Intelligence:** Users in the TrailMap group frequently used words such as “smart”, “intuitive”, and “adaptive” to describe the matching process. One participant commented, “*It felt like after a week, the system learned who was good at giving advice and put me in touch with them.*” [P18]. In contrast, control group users often described their matches as “*random*” or “*a lottery*” *[P61]*.
- **Increased Trust:** TrailMap participants reported a higher degree of trust in the platform’s ability to provide a useful connection. This perception of reliability and intelligence appears to foster greater willingness to engage and share. This aligns with the design principles, suggesting that system predictability and effectiveness are key drivers of user trust in health technologies [47,48,49,50,51,52].
- **Emergent Mentorship:** A network analysis of the interaction graph from the TrailMap group (visualised in Figure 6) revealed the spontaneous emergence of several distinct helper clusters. These were small groups of highly rated users who accumulated strong pheromone trails and became de facto community mentors, frequently receiving requests from a wide range of seekers without any formal designation or top-down curation. This emergent social structure was absent from the random graph of the control group.

## 5. Discussion

### 5.1. Principal Findings: From Efficiency to Ecosystem Sustainability

This study provides robust, converging evidence from both simulation and a human-centered trial that a biomimetic algorithm inspired by ant foraging can significantly improve the performance of a digital peer support system. Thus, the primary hypotheses were strongly supported. The TrailMap algorithm led to a substantial reduction in wait times for helpful support and a significant increase in the perceived quality of interactions.

However, the most significant contribution of this study lies beyond mere efficiency gains. The core problem confronting online support communities is sustainability, driven by a vicious cycle of inefficient matching, participation inequality, and helper burnout [5,6,18,19]. Our results demonstrate that TrailMap directly addresses this cycle by regulating key system-level dynamics, such as workload distribution and participation equity. The quantitative evidence for this regulatory capacity is clear. By measuring the distribution of workload with the Gini coefficient, we showed that TrailMap creates a more equitable system (reducing the coefficient from 0.58 to 0.34). This reduction in the Gini coefficient is not an incidental outcome, but rather, an emergent property arising directly from two core coupled mechanisms in the algorithm’s design. The first is the stochastic path selection rule (Section 2.3), which inherently promotes exploration by ensuring that even less-established helpers receive some traffic, thus preventing the system from becoming overly greedy and fixated on top performers. The second, and equally critical, mechanism is the pheromone evaporation rule (Section 2.4), which acts as a systemic counter-force to the entrenchment of power-law dynamics, actively working against the indefinite accumulation of pheromones on a few elite helpers who might otherwise become overworked “super-helpers” [53]. By spreading the load, TrailMap fosters a more resilient and sustainable community structure, reducing the risk of collapse owing to the burnout of a few key members. The qualitative finding of ‘emergent mentorship’ (Section 4.2, Figure 6) is a clear demonstration of stigmergic self-organisation. The system does not require the top-down assignment of roles, privileges, or mentor status. Instead, the community collectively identifies and reinforces paths to effective helpers through the decentralised mechanism of pheromone deposition. This bottom-up process, where agents coordinate indirectly by modifying a shared environment, creates an emergent social structure optimised for support, which is a hallmark of well-functioning complex adaptive systems. The convergence of the simulation and pilot study results strongly supports the real-world applicability of this approach.

An important aspect of TrailMap’s design is how it transforms a classic computational challenge—the ‘cold start’ problem—into a feature for social sustainability. In conventional recommender systems, the inability to recommend new items lacking ratings is a significant flaw that needs to be overcome. Similarly, in ACO, the initial random walk of the ants before pheromone trails are established is a necessary, but inefficient, first step. However, TrailMap leverages this initial state. The inclusion of the $\tau_{min}$ constant in the path selection formula (Section 2.3) guarantees that every new or low-activity helper has a nonzero probability of being selected. This ’enforced exploration’ is the system’s explicit mechanism for onboarding new contributors. It actively prevents the community from calcifying a small group of established helpers, ensuring a continuous influx of new participants, and distributing opportunities for engagement. In this context, ‘cold start’ is not a problem to be solved, but a vital, perpetual mechanism for community renewal and resilience.

### 5.2. TrailMap as Problem-Driven Biomimicry

TrailMap design serves as a clear example of the problem-driven paradigm of biomimicry [54,55]. Biomimetic design processes are typically categorised into two approaches: solution-driven or problem-driven [56,57,58]. In the solution-driven approach, a fascinating biological mechanism is first identified, and the designer then seeks problems to which it can be applied. In contrast, the problem-driven approach, which guided this study, began with a well-defined human or technical challenge [54].

The design process did not begin with biological curiosity, but with a concrete system- level problem of creating sustainable digital peer support communities. First, we identified specific failure modes: inefficient routing, load imbalance, and helper burnout. Only then did we search the biological world for analogues of systems that solve similar abstract problems of decentralised, adaptive resource allocation. The ant colony, with its stigmergic communication system, is a compelling analogue [21,22]. We followed the formal abstraction process described by Helms et al. [54], described as follows:

- Biological Analogue: An ant colony seeking efficient paths to food sources in a dynamic environment;
- Technological Problem: A help-seeker in a digital community seeks an efficient path to a helpful peer in a dynamic social environment;
- Abstraction and Transfer: We systematically mapped the core principles of a biological system to our computational framework, as follows:
  – Ants → Peer users (seekers and helpers)
  – Pheromone Trail → A numerical weight (*τ*) on a user-interaction path
  – Pheromone Deposition → User-provided helpfulness ratings $\left(R_{h}\right)$
  – Pheromone Evaporation → A time-based exponential decay function (*ρ*)
  – Probabilistic Path Following →A stochastic routing algorithm biasing seekers towards high-pheromone helpers.

**Figure 7:**
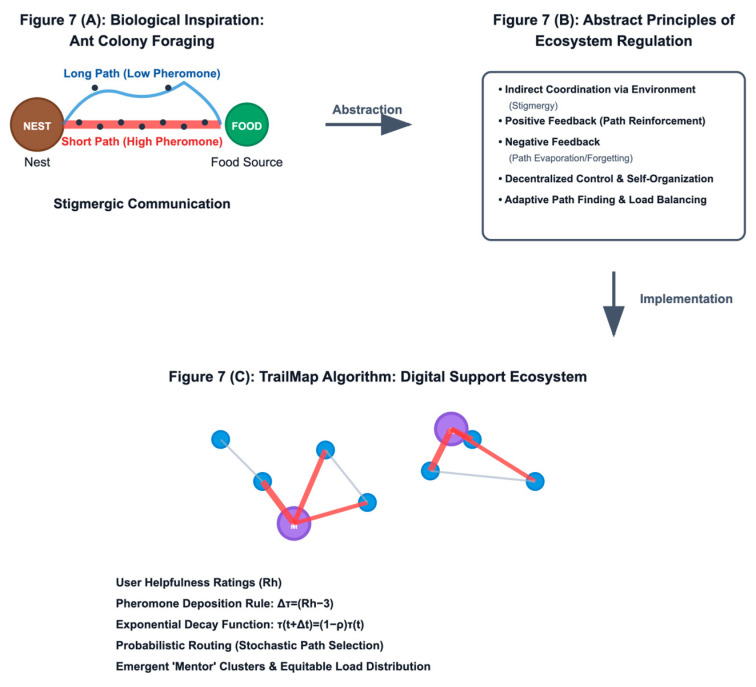
The biomimetic transfer process for TrailMap illustrates the flow from the concrete biological system (**A**) through the abstraction of its core regulatory principles (**B**) to the final computational implementation within the digital peer support platform (**C**). This process highlights the focus on transferring functions and ecosystem dynamics, not merely form.

This deliberate, problem-first methodology is visually summarised in Figure 7, which illustrates the complete transfer process from biological inspiration to computational implementation. The process begins with the observation of a natural system, abstracts its core functional principles, and then translates these principles into a specific algorithmic solution tailored to the technological problem domain, the performance of which was subsequently validated through a two-phase study.

This mapping transcends mere metaphors to become a systematic transfer of functional components, as detailed in Table 5. This formal breakdown clarifies how each element of the ant colony’s foraging strategy is functionally realised within the TrailMap algorithm. Through this lens, the core biomimetic insight becomes clear: the goal is not simply to replicate how ants find short paths but to import the entire colony’s capacity to create a resilient, adaptive, and self-organising system for resource discovery and allocation, thereby transferring these higher-order ecosystem properties to the digital community. This approach contributes a novel paradigm for human–computer interaction design, demonstrating how algorithmic systems inspired by natural processes can enhance the equity, adaptivity, and resilience of digital health platforms.

### 5.3. Ethical Implications and Algorithmic Governance

Deploying an autonomous optimisation algorithm in the sensitive domain of mental health necessitates a profound consideration of the ethical risks involved, such as the potential to amplify harm, enable user manipulation, or create new forms of social exclusion. TrailMap is a form of “societal algorithmic mediation”, as it actively shapes social interactions and access to support [59]. While designed for benevolent outcomes, any value-laden algorithm is susceptible to unintended consequences and biases [59]. Without sufficient safeguards, the algorithm can inadvertently amplify harm by reinforcing the pheromone trails of a user who is highly engaging but provides unsubstantiated or detrimental advice. Therefore, the algorithm must be embedded within a robust ethical governance framework. We have incorporated several essential guard rails into our design.

- **The ’Negative Reinforcement Mechanism Guard-Rail:** The pheromone deposition rule, $∆\tau_{sh}={(R}_{h}-3)$, is a critical safety feature. This allows the community to collectively and dynamically suppress harmful content or actors. When users are rated as unhelpful or providing bad advice, they accumulate negative pheromones, making the system actively learn to avoid them. This provides a decentralised, bottom-up mechanism to address the pervasive challenge of misinformation in online health communities [6,60], a challenge that has become increasingly critical as social platforms grapple with their dual role as sources of both support and harmful polarisation [61,62].
- **Human-in-the-Loop Moderator Pruning:** The algorithm is designed to augment, not replace, human oversight. The system should feature a moderator dashboard that visualises high-pheromone trails and flags users who, despite high ratings, may be exhibiting problematic behaviour (e.g., monopolising conversations). This allows human moderators to focus their attention efficiently where needed, addressing the challenge of moderator overload in large communities [63,64]. Moderators must have the power to manually inspect, “prune”, or completely reset the pheromone trails of any user identified as spreading misinformation or engaging in predatory behaviour, effectively removing them from the trusted helper pool.
- **Transparency and Explainable AI (XAI):** User trust is paramount for engagement and disclosure in digital health settings [47,49,50,51,52]. To foster this trust, the system’s operations should not be an opaque “black box” [65]. While the full probabilistic model may be too complex to display, the system should provide simple, clear explanations for its matches, aligning with the principles of XAI. For example, a user could be shown a message like, “You were connected with Jane because she has been rated as very helpful by others recently”. This transparency respects user agency and builds confidence in the platform’s integrity.

### 5.4. Limitations

This study has several limitations that must be acknowledged. First, the pilot study was conducted with a relatively small (N = 71) and homogenous sample, as detailed in our participant demographics (Section 3.2, Table 2). While this is appropriate for a feasibility and validation study, the findings require replication in larger, more diverse, and clinical populations to establish generalisability.

Second, the four-week duration of the pilot study limited the assessment of long-term effects. This timeframe was sufficient for observing immediate gains in efficiency and perceived support quality, but it is too short to draw firm conclusions about slower-developing phenomena, such as helper burnout or the evolution of community social structures. A longitudinal study spanning several months is necessary to fully observe these crucial dynamics.

Third, the current implementation of TrailMap is content agnostic. It relies solely on user-provided helpfulness ratings and has no understanding of the semantic content of support requests or replies. This means that it cannot distinguish between a request about anxiety and one about relationship stress, nor can it match users based on topic similarity. This is a significant limitation that reduces the potential specificity of matching. Finally, while the agent-based simulation was invaluable for parameter tuning and understanding the algorithm’s core dynamics, it is necessarily an abstraction of complex and nuanced human behaviour.

A key limitation of our agent-based simulation is its reliance on a predefined agent distribution, specifically an 80/20 split between help-seekers and helpers. This ratio was deliberately chosen to model the well-documented “participation inequality” phenomenon, often cited as the “1% rule” or the “90-9-1 principle”, where a small minority of users contribute the vast majority of content or support in online communities [5,11,12]. Our findings, which demonstrate TrailMap’s significant advantages in efficiency and load balancing, are therefore most applicable to platforms that exhibit this characteristic helper scarcity. It is plausible that in a system with a different dynamic, such as a reversed ratio with an abundance of helpers (e.g., 20% seekers vs. 80% helpers), the core problems of helper burnout and inefficient matching would be greatly diminished. In such a scenario, the sophisticated routing mechanism of TrailMap might offer little to no advantage over simpler random allocation algorithms, and its contribution would be less pronounced. Consequently, the generalisability of our current findings is bounded by this assumption, and future work should explore the algorithm’s performance across a spectrum of community compositions.

## 6. Future Research and Conclusions

### 6.1. Future Directions for Digital Therapeutics and HCI

Building on the demonstrated benefits of TrailMap in terms of response efficiency and load equity, several avenues for future research emerge that can advance both digital therapeutics and the design of intelligent human–computer interaction systems.

One important direction is the integration of natural language processing (NLP) to overcome TrailMap’s current content-agnostic limitation. Incorporating topic modelling and sentiment analysis could enable the system to identify the emotional tone and subject matter of a help-seeker’s request (e.g., anxiety, grief, stress), and to match them with helpers who have demonstrated effectiveness in those specific areas. This would enhance the semantic precision of peer matching and improve the therapeutic alliance.

Another critical area involves investigating user trust and perceptions of algorithmic mediation. Future studies should investigate how varying levels of transparency and explainability in system operations affect user engagement, perceived fairness, and psychological comfort. Mixed-methods approaches combining validated trust metrics and qualitative interviews could shed light on how users respond to probabilistic decision-making in sensitive mental health contexts.

Finally, an emerging research frontier is the development of hybrid human–AI peer support systems. These would combine human helpers with conversational agents capable of providing immediate factual support. Routing mechanisms can be designed to intelligently allocate requests based on the nature of the query, for example, directing emotionally complex or empathetic conversations to humans and more informational requests to AI companions. Research in this direction would need to address not only optimisation strategies but also the dynamics of collaboration between human and artificial agents in shared digital ecosystems.

### 6.2. Conclusions

This study introduces and validates TrailMap, a novel biomimetic algorithm for digital peer support, grounded in the principles of ant colony optimisation. Through a combination of agent-based simulation and a human-centered pilot study, we demonstrated that TrailMap significantly improves the efficiency of support delivery while also promoting a more equitable and resilient community structure.

By abstracting biological mechanisms such as stigmergy, pheromone deposition, and evaporation into a socially aware computational framework and embedding these within an ethically guided system design, we show that nature-inspired approaches can yield adaptive, scalable, and human-centric solutions to persistent challenges in digital health and HCI.

TrailMap represents a promising step towards the development of self-regulating online communities that can sustain participation, prevent helper burnout, and provide effective and timely mental health support. Future work should focus on extending its content sensitivity, enhancing transparency and user trust, and exploring its integration within hybrid human–AI ecosystems for next-generation digital therapeutics.

## Figures and Tables

**Table 1:** Formal Parameters of the Agent-Based Simulation Model.

Parameter	Symbol	Value/Distribution	Description
Total Agents	*N*	1000	The total number of individual agents in the simulated community.
Seeker Proportion	$P_{seeker}$	0.2	The fixed proportion of agents actively seeking help at any given time.
Helper Proportion	$P_{helper}$	0.8	The fixed proportion of agents available to provide help.
Latent Helpfulness	$H_{latent}$	Beta (α = 2, β = 5)	An intrinsic helpfulness score for each helper, drawn from a right-skewed Beta distribution to model a community with fewer exceptionally effective helpers.
Helpfulness Rating	$R_{h}$	Discrete distribution (μ = $H_{latent}$) + Gaussian noise	The rating a seeker provides, generated stochastically with the helper’s latent score as the mean, plus noise to simulate subjective perception.
Simulation Duration	$\tau_{sim}$	720 h (30 days)	The total virtual time for which each simulation run was executed.
Time Step	Δt	1 h	The discrete interval at which system-wide pheromone evaporation was calculated.

**Table 2:** Summary of Pilot Study Participant Demographics (N = 71).

Characteristic	Subcharacteristic	TrailMap Group (*n* = 36)	Control Group (*n* = 35)	Total (N = 71)
Age (years)	Mean (SD)	22.4 (2.8)	22.1 (2.5)	22.3 (2.6)
Gender	Woman	20 (55.6%)	19 (54.3%)	39 (54.9%)
	Man	15 (41.7%)	15 (42.9%)	30 (42.3%)
	Nonbinary/Other	1 (2.8%)	1 (2.9%)	2 (2.8%)
Previous Peer Support Experience	Yes	9 (25.0%)	8 (22.9%)	17 (23.9%)
	No	27 (75.0%)	27 (77.1%)	54 (76.1%)

**Table 3:** Key Performance Indicators from Agent-based 30 Virtual Days Simulation.

Condition	Mean Time to Helpful Response (min)	Gini Coefficient of Load Distribution
Random Routing (Control)	84	0.58
TrailMap (48 h half-life)	24	0.34

**Table 4:** Statistical Comparison of Pilot Study Outcomes (N = 71).

Outcome Measure	TrailMap Group (*n* = 36)	Control Group (*n* = 35)	Test Statistic, *p*-Value	*p*-Value
Median Wait Time (min)	19 (IQR: 12–31)	82 (IQR: 45–124).	*U* = 217	*p* < 0.001
Mean Helpfulness Rating	4.31 (SD: 0.68)	3.45 (SD: 1.02)	*t*(69) = 4.51	*p* < 0.001

**Table 5:** Formal Mapping of the Biological Analogue to the TrailMap System.

Biological System Component (Ant Colony)	Abstract Principle	TrailMap Implementation (Online Support Ecosystem)
Ant colony seeking food	A community seeking a distributed resource	A peer support community seeking helpful interactions.
Individual ants	Autonomous agents with simple rules	Individual users (seekers and helpers).
Physical environment (ground)	A shared medium for indirect communication	The platform’s interaction graph and digital space.
Pheromone chemical	A volatile, quantitative trace of positive interaction	A numerical weight, *τ*, on a potential seeker-helper path
Pheromone deposition by ants	Stigmergic reinforcement based on success	Deposition of ‘digital pheromones’ ∆*τ* based on user-provided helpfulness ratings ${(R}_{h}).$
Pheromone evaporation over time	Forgetting mechanism for adaptivity and dynamism	Exponential decay of τ over time, allowing the system to forget inactive or unhelpful users.
Ants’ probabilistic path following	Stochastic exploration biased by trace strength	A probabilistic routing algorithm that biases seekers towards helpers with high *τ* but still allows for the exploration of new helpers.
Discovery of shortest paths	Emergent optimisation of efficiency	Reduction in wait time for a helpful response.
Dynamic switching between food sources	System adaptiveness and resilience	Dynamic re-routing away from burned-out/inactive helpers and towards newly active and effective helpers
Collective foraging behaviour	Self-organising load balancing	Equitable distribution of support requests (lower Gini coefficient), mitigating the ’super-helper’ phenomenon.

## Data Availability

The datasets presented in this article are not readily available because they are part of ongoing studies and following an embargo from the date of publication to allow for commercialization of research findings.
